# Caspase Inhibitors of the P35 Family Are More Active When Purified from Yeast than Bacteria

**DOI:** 10.1371/journal.pone.0039248

**Published:** 2012-06-14

**Authors:** Ingo L. Brand, Srgjan Civciristov, Nicole L. Taylor, Gert H. Talbo, Delara Pantaki-Eimany, Vita Levina, Rollie J. Clem, Matthew A. Perugini, Marc Kvansakul, Christine J. Hawkins

**Affiliations:** 1 Department of Biochemistry, La Trobe Institute for Molecular Science, La Trobe University, Bundoora, Victoria, Australia; 2 Department of Biochemistry and Molecular Biology, Bio21 Molecular Science and Biotechnology Institute, The University of Melbourne, Parkville, Victoria, Australia; 3 Division of Biology, Kansas State University, Manhattan, Kansas, United States of America; 4 Murdoch Children's Research Institute, Royal Children's Hospital, Parkville, Australia; University Freiburg, Germany

## Abstract

Many insect viruses express caspase inhibitors of the P35 superfamily, which prevent defensive host apoptosis to enable viral propagation. The prototypical P35 family member, AcP35 from *Autographa californica* M nucleopolyhedrovirus, has been extensively studied. Bacterially purified AcP35 has been previously shown to inhibit caspases from insect, mammalian and nematode species. This inhibition occurs via a pseudosubstrate mechanism involving caspase-mediated cleavage of a “reactive site loop” within the P35 protein, which ultimately leaves cleaved P35 covalently bound to the caspase's active site. We observed that AcP35 purifed from *Saccharomyces cerevisae* inhibited caspase activity more efficiently than AcP35 purified from *Escherichia coli*. This differential potency was more dramatic for another P35 family member, MaviP35, which inhibited human caspase 3 almost 300-fold more potently when purified from yeast than bacteria. Biophysical assays revealed that MaviP35 proteins produced in bacteria and yeast had similar primary and secondary structures. However, bacterially produced MaviP35 possessed greater thermal stability and propensity to form higher order oligomers than its counterpart purified from yeast. Caspase 3 could process yeast-purified MaviP35, but failed to detectably cleave bacterially purified MaviP35. These data suggest that bacterially produced P35 proteins adopt subtly different conformations from their yeast-expressed counterparts, which hinder caspase access to the reactive site loop to reduce the potency of caspase inhibition, and promote aggregation. These data highlight the differential caspase inhibition by recombinant P35 proteins purified from different sources, and caution that analyses of bacterially produced P35 family members (and perhaps other types of proteins) may underestimate their activity.

## Introduction

Members of the caspase family of cysteine proteases are key regulators of insect cell death [1]. Insect viruses of the baculovirus [2] and entomopoxvirus [3] families have evolved to express caspase inhibitors of the P35 family, which suppress the defensive host apoptosis that their infection would otherwise trigger [4]. The first identified and best studied P35 family member is AcP35, from *Autographa californica (Ac)* M nucleopolyhedrovirus (MNPV) [5]. AcP35 possesses an extremely broad specificity, potently inhibiting caspases from mammals and nematodes in addition to insects [6]–[11]. Only a few caspases, such as *Drosophila* DRONC [12], [13], its putative *Spodoptera* ortholog Sf-caspase-x [14] and human caspase 9 [15] have been reported to withstand the inhibitory activity of AcP35. Biochemical and structural analyses of bacterially-purified AcP35 have defined its mechanism of action [16]–[19]. Caspase cleavage of AcP35 within the so-called “reactive site loop” (RSL) provokes a major conformational change that places the amino terminus of AcP35 into the active site of the caspase, where it prevents hydrolysis of a thioester bond between the inhibitor and the protease [16]–[19]. This traps the caspase in a P35-bound form [16] incapable of cleaving substrates. Although AcP35 inhibits a broad range of caspases, and insect caspases are its natural targets, AcP35 was also found to potently inhibit the bacterial cysteine protease gingipain-K, via a similar suicide substrate mechanism to that used by AcP35 to inhibit caspases [20].

A number of other baculoviruses encode close relatives of AcP35. We recently reported that one of these sub-family members, MaviP35 from *Maruca vitrata* MNPV [21], shared a caspase inhibitory mechanism with AcP35 but exhibited somewhat different specificity [22]. Some baculoviruses [23], [24] encode more distant relatives of AcP35, including members of the “P49” sub-family. SpliP49 from *Spodoptera littoralis* NPV inhibited a broad range of caspases, via a similar mechanism to AcP35, but unlike AcP35 could target insect initiator caspases [7], [25]–[28]. *Amsacta moorei* entomopoxvirus encodes another distant AcP35 relative, P33. Despite sharing only 25% amino acid identity with AcP35, P33 inhibited apoptosis, bore a similar specificity profile to AcP35, and mutational analyses suggested it employed a similar mechanism of action [3].

Previous biochemical analyses of the caspase inhibitory mechanism, potency and specificity of AcP35 and P33 have utilized C-terminally His_6_-tagged bacterially-purified proteins [3], [6]–[10], [16]–[20], [25], [29]–[33]. Recombinant His_6_-tagged SpliP49 was initially purified from Sf-21 insect cells [25], due to insolubility in bacteria. Subsequent inclusion of an unstructured linker between SpliP49 and the tag enabled purification of soluble SpliP49 from *E. coli*, which apparently exhibited similar activity to the protein isolated from insect cells [27].

We recently compared the activities and specificities of AcP35 and MaviP35 [22]. Here we report that, although AcP35 and MaviP35 could be produced as abundant soluble proteins by *Saccharomyces cerevisiae* and *Escherichia coli*, both proteins were more potent caspase inhibitors when purified from yeast rather than bacteria. This study explored the molecular basis for this differential potency.

## Results

### Enhanced activity of AcP35 and MaviP35 following purification from yeast

**Figure 1:**
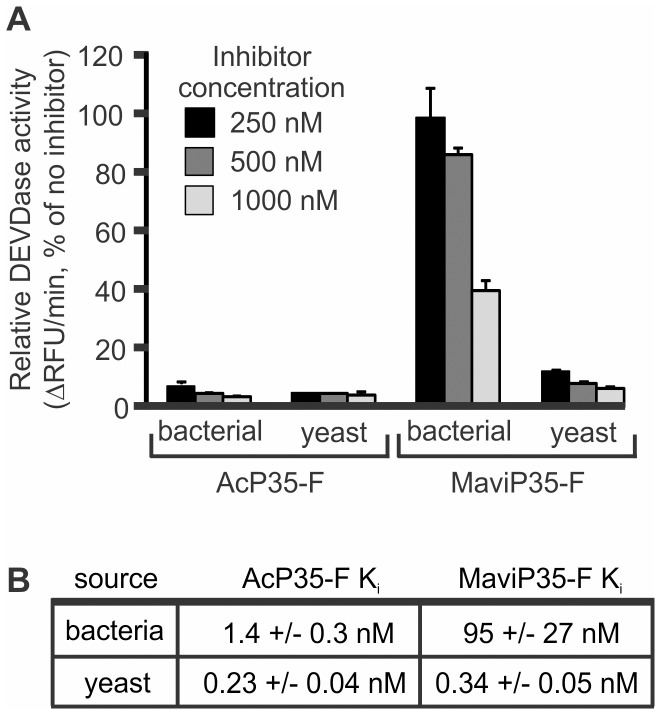
MaviP35 and AcP35 are more active when purified from yeast than bacteria. (**A**) FLAG-tagged MaviP35 or AcP35 were purified from bacteria or yeast. The indicated concentrations of inhibitors were assayed for their ability to inhibit cleavage of 100 µM Ac-DEVD-AFC by 30 nM caspase 3. Error bars represent S.E.M. from three independent replicates. (**B**) A competitive model was used to determine the caspase 3 inhibition constants for the P35 proteins purified from yeast [22] and bacteria.

Sequencing of the *Mavi*MNPV genome [21] revealed that this virus encoded a P35 orthologue that was 81% identical to the founding member of this family, AcP35, and modeling suggested they may adopt similar structures [22]. MaviP35 was able to inhibit both insect and mammalian cell death, confirming that it can act as an apoptosis inhibitor [22]. To explore the caspase inhibitory properties of AcP35 and MaviP35, we initially purified the viral apoptosis inhibitors as C-terminally FLAG-tagged proteins from *Escherichia coli*. In three separate experiments, bacterially produced AcP35 potently inhibited the proteolytic activity of caspase 3, but MaviP35 only partially impaired the ability of caspase 3 to cleave a peptide substrate (data not shown). This inefficiency was puzzling, as we had found that AcP35 and MaviP35 conferred similar protection against yeast death triggered by expression of active caspase 3 [22]. This discrepancy suggested to us that the MaviP35 expressed in yeast may be a more potent caspase inhibitor than MaviP35 expressed in bacteria. To directly test this hypothesis, we purified FLAG-tagged MaviP35 from *Saccharomyces cerevisiae*. This protein was dramatically more efficient at inhibiting caspase 3 than its bacterially produced counterpart (Figure 1A). Quantitation of caspase inhibition by MaviP35 purified from bacteria and yeast revealed that the protein purified from *E. coli* (Figure 1B) was around 300-fold less potent than that purified from yeast [22]. We observed a similar, but less dramatic, difference with AcP35. Yeast-purified AcP35 inhibited caspase 3 [22] about six times more efficiently than bacterially-purified AcP35 (Figure 1B).

### Analyses of primary structures of P35 proteins purified from bacteria and yeast

**Figure 2:**
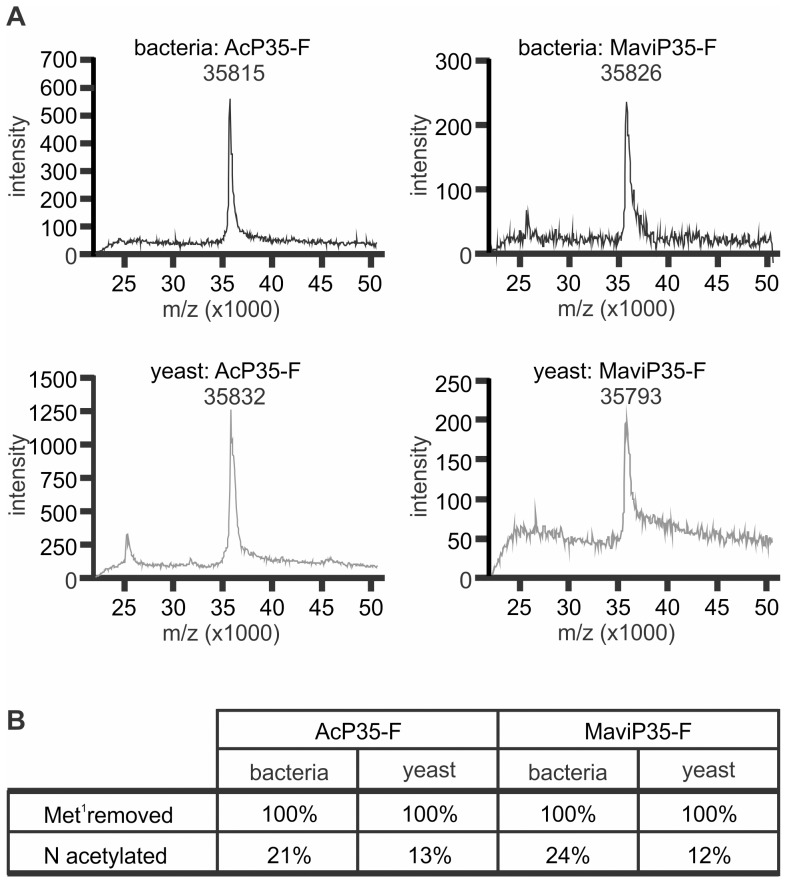
Expression in bacteria or yeast does not substantially alter the primary structural features of MaviP35 and AcP35. FLAG-tagged MaviP35 and AcP35 were purified from bacteria or yeast. (**A**) Each sample was analyzed using Matrix Assisted Laser Desorption Ionisation-Mass Spectrometry. (**B**) Reduced and alkylated proteins were subjected to trypsin digestion, then the peptides were analyzed by LC-ESI-MS. The intensity of peaks of masses corresponding to the predicted peptide mass of unmodified or modified amino terminal tryptic peptides were measured. Based on the assumption that the peak intensities are proportional to the peptide amount, the relative amount of each peptide was calculated. The integrities of the peptides were confirmed by MS/MS analysis.

A number of possible explanations were tested to explore the differential activities of P35 family proteins isolated from bacteria and yeast. Mass spectrometry methods were employed to examine the primary structures of AcP35 and MaviP35 purified from the different sources, to determine whether post-translational modifications differed depending on the source of recombinant protein. The proteins from both sources had molecular weights consistent with each protein lacking its initiating methionine residue (Figure 2A). This excluded the possibility that prokaryotic proteases cleaved and incapacitated the bacterially-purified inhibitors, or that large post-translational modifications which modulated function occurred in one species but not the other. Analysis of the amino terminal tryptic peptides generated from each protein confirmed the absence of the initiating methionine (Figure 2B). Similar proportions of bacterially purified AcP35 and MaviP35 were N-acetylated (21% and 24% respectively). In contrast, only 13% of AcP35 and only 12% of MaviP35 proteins were N-acetylated when expressed in yeast. This difference of around 10% between the proteins purified from bacteria and yeast seems unlikely to account for the 280-fold superiority of yeast-purified MaviP35 with respect to caspase inhibition (Figure 1B).

### Analyses of secondary structures of MaviP35 purified from bacteria and yeast

**Figure 3:**
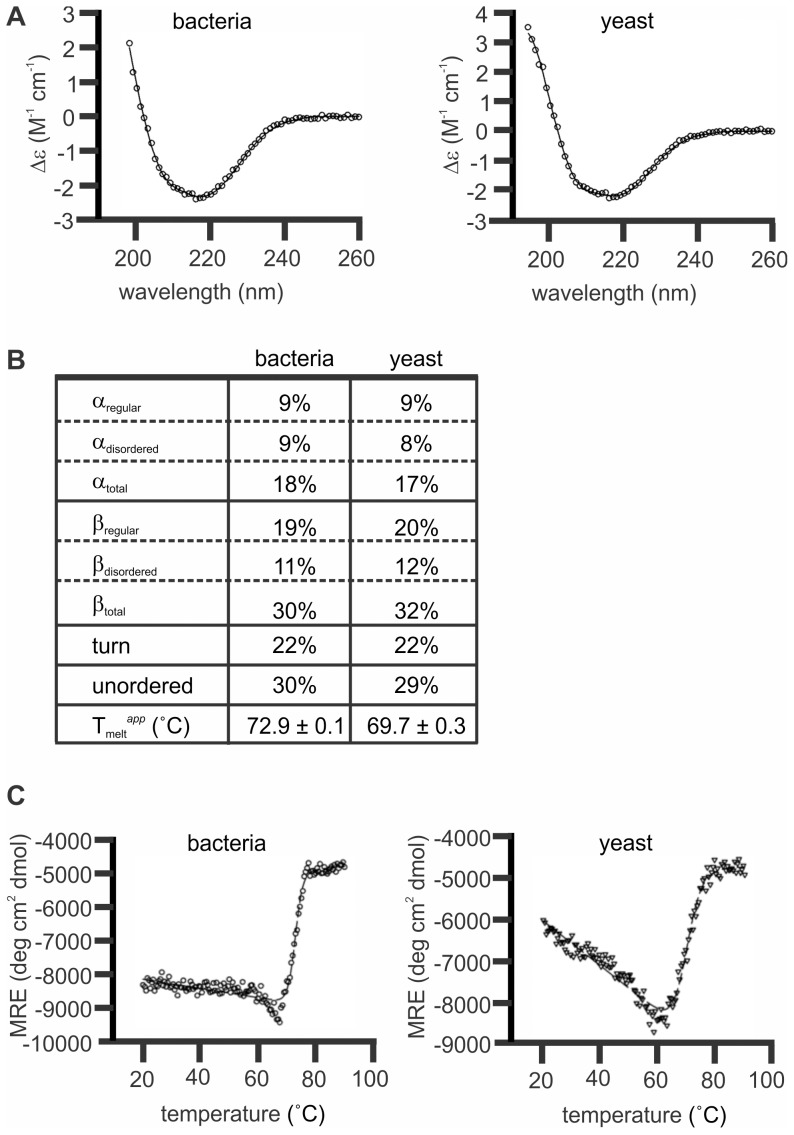
MaviP35 proteins purified from bacteria and yeast have similar secondary structures. FLAG-tagged MaviP35 proteins were purified from yeast and bacteria and then subjected to circular dichroism (CD) analyses. (**A**) Wavelength scans were performed at 20°C. The final spectra is the average result of three scans (open circles). The CONTINLL algorithm calculated the nonlinear least squares best fit (solid line) against the SP29 protein database with r.m.s.d. values≤0.073. (**B**) Table of secondary structure proportions and apparent melting temperature for MaviP35 purified from bacterial and yeast. (**C**) Ellipticity at 216 nm was measured between 20 and 90°C (open circles). The nonlinear regression analysis (dashed lines) fitted the curves to a one step transition between folded and unfolded confirmations.

The above-mentioned mass spectrometry analyses suggested that the P35 proteins expressed in bacteria and yeast had similar primary structures. Circular dichroism (CD) spectroscopy was used to explore the possibility that differences in secondary structure may underlie the substantially greater activity exhibited by yeast-purified MaviP35, relative to bacterial MaviP35. Wavelength scans of each sample were performed to estimate the fractions of various secondary structural elements adopted by bacterially and yeast-expressed MaviP35 in aqueous solution. The resulting spectra each displayed a single minimum at approximately 216 nm (Figure 3A, open circles), which is indicative of a protein sample with predominantly β structure [34]. The CD spectra were fitted by nonlinear least squares regression against relevant protein databases [35], [36]. The best fit for the MaviP35 samples (Figure 3A, solid line) was obtained by applying the CONTINLL algorithm against the SP29 database. This predicted that both samples had large proportions of β-structure (30–32%) and unordered secondary structure (29–30%) in combination with significant proportions of β-turn (22%) and α-helical (17–18%) structure (Figure 3B). These secondary structure proportions correlate well with structural predictions for MaviP35 [22] based on the structure of AcP35 determined by X-ray crystallography [18]. To assess thermal stability of the samples, CD thermal denaturation studies were performed at 216 nm between 20°C and 90°C (Figure 3C, open symbols). Nonlinear regression analysis of the data to a one step transition model resulted in a fit with R^2^ = 0.9843 and 0.9737 for the bacterial and yeast samples respectively (Figure 3C, dashed lines). These fits revealed the apparent melting temperatures (T_melt_ *^app^*) for bacterially-produced MaviP35 to be 72.9±0.1°C and yeast-isolated MaviP35 to be 69.7±0.3°C, suggesting that the bacterially-purified form of MaviP35 was slightly more thermostable than the yeast-purified form.

### Comparisons of the oligomeric status of MaviP35 isolated from bacteria and yeast

**Figure 4:**
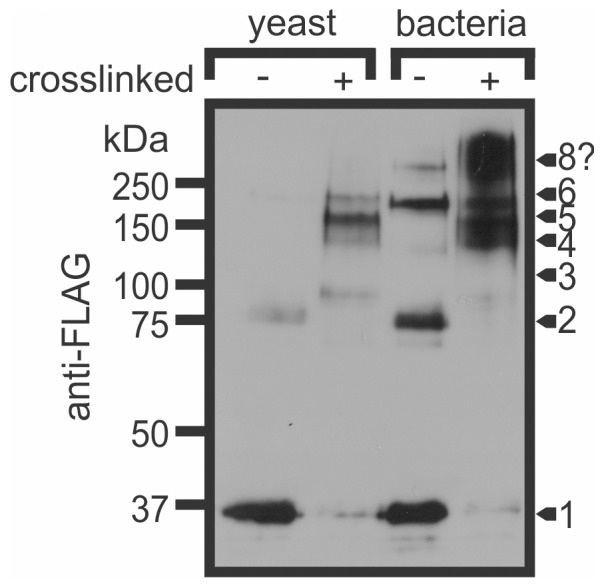
Bacterially produced MaviP35 is more prone to aggregation *in vitro* than yeast-expressed MaviP35. FLAG-tagged MaviP35 proteins were purified from bacteria or yeast and then treated with 0 or 5 µM of the crosslinker BMH prior to analysis by SDS-PAGE and anti-FLAG immunoblotting. Arrows on the right indicate the expected positions of various oligomeric species based on the migrations of the molecular weight markers. The expected molecular weight of octameric MaviP35 (286 kDa) would be greater than that of the largest marker used, so its migration is difficult to accurately estimate. A representative immunoblot is shown from four separate experiments.

Subsequent experiments further probed the possibility that species-dependent conformational differences may account for the differential potencies of MaviP35 purified from yeast and bacteria. Chemical crosslinking revealed differences in the oligomeric status of MaviP35 proteins isolated from bacteria and yeast (Figure 4). Crosslinking of MaviP35 isolated from yeast revealed a major species that migrated as expected for a pentamer, and less abundant apparent tetrameric and hexameric forms. The majority of the bacterially-purified MaviP35 proteins migrated as monomers when subjected to reducing SDS-PAGE. However, apparent dimers and hexamers were also present despite samples being boiled in loading dye containing β-mercaptoethanol prior to electrophoresis. After crosslinking, the migration of the bacterially-produced MaviP35 protein suggested the existence of oligomers composed of four to ten monomers (Figure 4).

**Figure 5:**
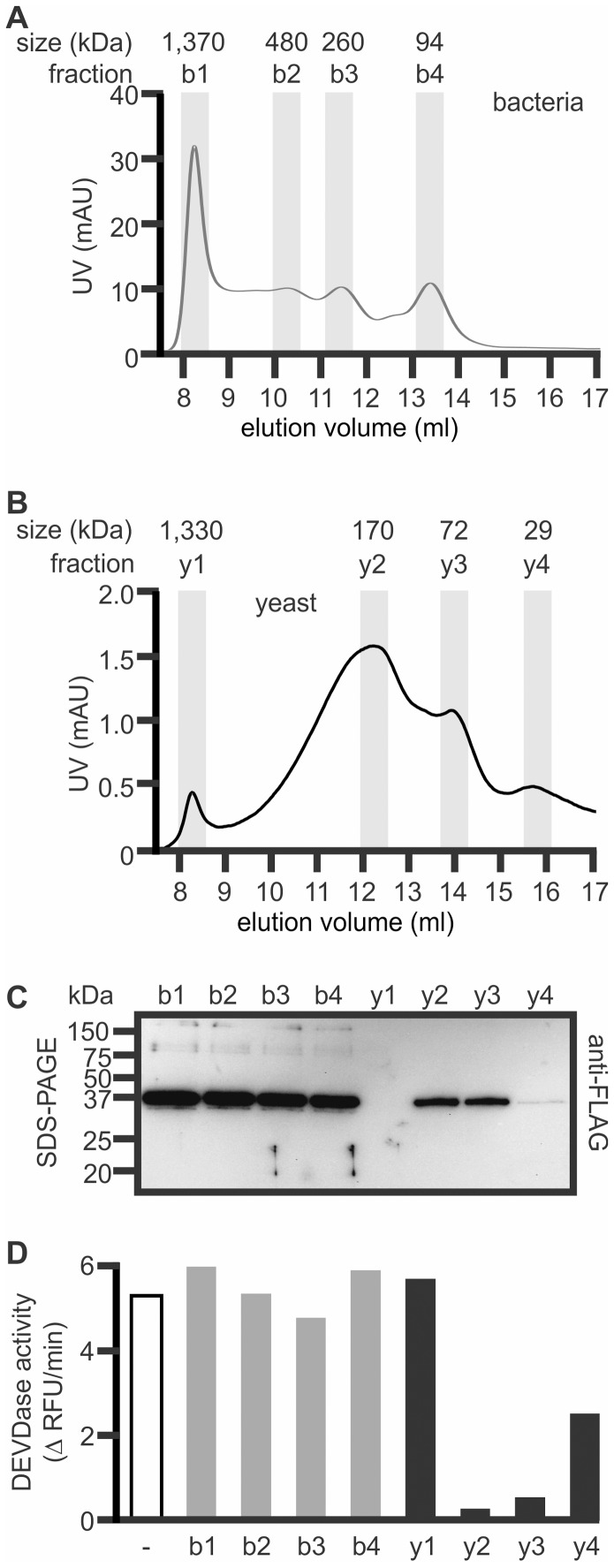
MaviP35 purified from yeast and bacteria differ in their mobility during size exclusion chromatography. FLAG-tagged MaviP35 proteins were purified from bacteria (**A**) or yeast (**B**) and then subjected to gel filtration. (**C**) Selected fractions were analyzed by SDS-PAGE and anti-FLAG immunoblotting. (**D**) Proteins from each selected gel filtration fraction or buffer (−) were mixed with caspase 3 and then maximal rate of cleavage of Ac-DEVD-AFC was monitored fluorometrically. (**C, D**) Because the yield of the yeast-purified protein was lower than the bacterial sample, the bacterial fractions were diluted 8-fold prior to these analyses.

MaviP35 proteins isolated from *E. coli* and *S. cerevisiae* were also subjected to gel filtration liquid chromatography (LC). The chromatograms of proteins isolated from the two sources were reproducibly different, throughout five separate experiments (Figure 5A, B, data not shown). Consistent with data from crosslinking experiments (Figure 4), none of the bacterially-produced protein migrated as expected for a monomer. Some of the bacterially-purified MaviP35 displayed migration consistent with a trimer, but a substantial amount of the protein eluted much earlier (Figure 5A), confirming the presence of the larger oligomers and aggregates observed following crosslinking and SDS-PAGE analysis (Figure 4). The yeast-isolated sample contained species with migration rates similar to those expected for monomers, dimers and pentamers (Figure 5B). In one experiment, fractions corresponding to the major gel filtration peaks were further analyzed. SDS-PAGE and anti-FLAG immunoblotting confirmed the presence of MaviP35 in each of the major bacterial fractions and the ∼170 kDa and ∼72 kDa fractions of the sample purified from yeast (Figure 5C). Consistent with the gel filtration chromatogram, very little monomeric MaviP35 was purified during gel filtration LC of the yeast-purified sample. The gel filtration LC fractions of MaviP35 purified from yeast that contained readily detectable protein (∼170 kDa and ∼72 kDa) efficiently inhibited caspase 3 activity (Figure 5D). In stark contrast, none of the fractions containing bacterially-produced MaviP35 affected the proteolytic activity of caspase 3 (Figure 5D), highlighting the poor activity of prokaryotically-expressed MaviP35.

**Figure 6:**
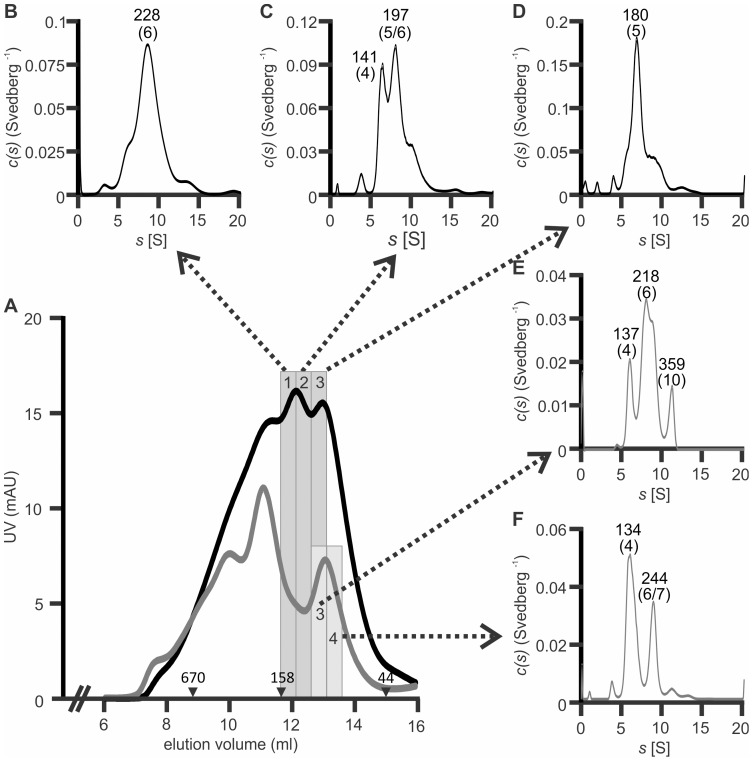
Analytical ultracentrifugation of MaviP35 purified from bacteria and yeast. (**A**) MaviP35-FLAG proteins were purified from bacteria (gray line) and yeast (black line) and then subjected to gel filtration. (**B–F**) Analytical ultracentrifugation of the fractions indicated in gray shading yielded the sedimentation profiles shown. The molecular weights estimated for each peak are stated. Numbers in parentheses indicate numbers of MaviP35-FLAG monomers that could comprise the major oligomeric species resolved by analytical ultracentrifugation.

To further investigate oligomeric status, gel filtration fractions of yeast- and bacterially-produced MaviP35 corresponding to 111–215 kDa and 89–138 kDa respectively were subjected to analytical ultracentrifugation. Sedimentation velocity analyses suggested the presence of predominantly tetrameric and hexameric species within the bacterially-purified sample (with minor dimeric and dodecameric species), whereas the majority of the yeast-isolated sample sedimented as expected for tetramers, pentamers and hexamers (with minor octameric species) (Fig. 6).

### Differential sensitivity to caspase cleavage of MaviP35 purified from bacteria versus yeast

**Figure 7:**
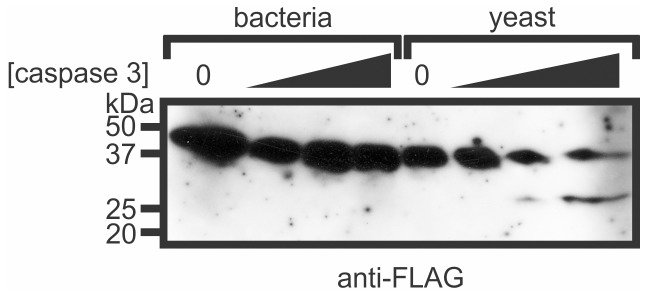
Caspase 3 can cleave MaviP35 purified from yeast but not bacteria. FLAG-tagged MaviP35 was purified from bacteria or yeast by affinity chromotography and gel filtration. The ∼94 kDa gel filtration fraction of the bacterial preparation (“b4” in Figure 5) and the ∼72 kDa fraction of the yeast-purified sample (“y3” in Figure 5) were incubated with 0, 10, 100 or 1000 ng/ml caspase 3 for 1 hour, and then subjected to SDS-PAGE and anti-FLAG immunoblotting.

The pseudo-substrate mechanism of inhibition employed by P35 proteins requires their cleavage by caspases. We reasoned that the conformation adopted by MaviP35 expressed in bacteria may obscure the reactive site loop and restrict its accessibility to caspases. Gel filtration fractions from the yeast and bacterial MaviP35 purifications were incubated with caspase 3 and their sensitivities to cleavage were monitored by immunoblotting. Although caspase 3 was able to cleave MaviP35 isolated from yeast to produce the expected 25.7 kDa carboxyl terminal FLAG-tagged product, it failed to cleave the bacterially-expressed protein (Figure 7).

## Discussion

This study revealed that the caspase inhibitory capacities of recombinant P35 proteins are influenced by the species used to express them. Expression in yeast yielded more potent caspase inhibitors than bacterial expression. The superiority of the yeast-purified AcP35, relative to the bacterially-produced form, was relatively minor. However, the difference in activities was dramatic in the case of MaviP35. Bacteria are convenient and effective for recombinant protein expression primarily because culturing is easy, inexpensive, and can achieve high yields [37]. Some proteins are not amenable to prokaryotic expression, either because the protein of interest tends to accumulate in an insoluble form within inclusion bodies, or because it requires eukaryotic post-translational modifications for activity [38]. In the case of these P35 family members, however, both AcP35 and MaviP35 were abundantly expressed as soluble proteins in *E. coli*. We explored the possibilities that differences in primary, secondary and quaternary structure may account for the differential potencies of the P35 proteins isolated from bacteria or yeast.

Because the amino terminus of AcP35 had previously been shown to be critical for its caspase inhibitory activity, we paid particular attention to potential species-specific post-translational modifications affecting this region of AcP35 and MaviP35. Unsuccessful attempts at amino-terminal sequencing of AcP35 previously led Riedl *et al* to speculate that the amino terminus of bacterially-produced AcP35 was a blocked methionine residue [19]. However, our mass spectrometry analyses suggested that AcP35 and MaviP35 isolated from both bacteria and yeast lacked initiating methionine residues. This removal, presumably mediated by methionine aminopeptidases [39], [40], was the only widespread post-translational modification observed, although it must be noted that subtle modifications which affect molecular weight very slightly may not have been detected using the mass spectrometry techniques we employed. Small proportions of the amino terminal tryptic peptides from each of the proteins were N-acetylated, but the source from which each protein was purified influenced this only marginally. Mass spectrometry analyses of intact proteins yielded the predicted molecular weights for full length proteins lacking the initiating methionine residues. This argues against the notions that bacterial proteases could cleave and inactivate P35 proteins expressed in *E. coli*, or that glycosylation or phosphorylation of the yeast-expressed P35 proteins could enhance their activity. This study directly compared the activities of C-terminally FLAG tagged P35 proteins isolated from bacteria and yeast, whereas most previous biochemical analyses of AcP35 have employed purified C-terminally His_6_-tagged proteins. The caspase inhibitory activities of FLAG- and His_6_-tagged P35 proteins were not compared, so additional research will be required to address the possibility that different epitope tags may differentially modulate the function of P35 proteins purified from different sources.

Circular dichroism spectroscopy was used to explore whether bacterial and yeast-purified MaviP35 differed in secondary structure. We concentrated on MaviP35 for these and subsequent analyses because its activity was more dramatically influenced than AcP35 by the species used for expression. Bacterial and yeast MaviP35 samples had similar secondary structure composition, with a large proportion of β-structure and unordered structure, and significant proportions of β-turn and α-helical structure. There were no discernible differences between the secondary structures of MaviP35 proteins purified from bacteria and yeast that could account for their markedly different activities. Thermal denaturation experiments revealed that both samples unfolded via a single state transition with respect to secondary structure, at 72.9±0.1°C and 69.7±0.3°C for the bacterial and yeast samples respectively. These data suggest that bacterially-produced MaviP35 is slightly, but significantly, more thermostable than the yeast purified protein, presumably because the proteins adopt different conformations and/or oligomeric states.

Previous studies have attributed various oligomeric states to AcP35. Dynamic light scattering suggested that bacterially-purified AcP35 formed dimers, whereas the crystal structure of AcP35 revealed a trimeric configuration [30]. In a separate study, AcP35 (bearing hemagglutinin and His_6_ tags) was purified from insect cells and observed to migrate during SDS-PAGE as expected for a trimer when crosslinked, but a dimer without crosslinking [31]. Yeast-two hybrid and mutagenesis analyses suggested that two molecules of AcP35 could dimerize via interactions between the helix α1 of one protein and the β-sheet of another [31]. In this study, we used gel filtration LC, chemical cross-linking and analytical ultracentrifugation to explore the oligomeric status of FLAG-tagged MaviP35 proteins purified from yeast and bacteria. Prokaryotically-expressed MaviP35 was more prone than the yeast-isolated protein to aggregate into higher order oligomers. However, this tendency seemed unlikely to account for the poor caspase inhibitory potential of this protein, because bacterially-expressed MaviP35 samples composed predominantly of tetrameric and hexameric species (isolated by gel filtration) exhibited negligible caspase inhibitory activity and were resistant to caspase cleavage. Yeast-purified MaviP35 also formed oligomers, but data from crosslinking and analytical ultracentrifugation studies suggested the presence of pentamers in addition to tetramers and hexamers. Gel filtration fractions containing these species possessed strong caspase inhibitory activity and were sensitive to caspase cleavage.

Considered together, our data show that bacterial and yeast expression produce MaviP35 proteins with slightly different solution properties that may reflect subtle differences in conformation. We postulate that yeast expression of MaviP35 yields a protein whose conformation permits access of a caspase to its reactive site loop, enabling cleavage and reorientation of the amino terminus to target the caspase's active site. This conformation predominantly assembles into tetramers, pentamers and hexamers. In contrast, the same protein expressed in bacteria at the same temperature folds into a more thermostable conformation that impedes caspase access to the cleavage site within the reactive site loop, and which forms tetramers and hexamers as well as higher order aggregates. These distinct conformational states do not appear to result from differential post-translational modifications, although we cannot rule out the involvement of modifications that only produce minor changes in molecular weight. Instead, it seems likely that different conformations result from well-described differences in folding mechanisms within bacterial versus yeast cells [38]. We previously observed that the C-terminal domain of thrombospondin-1 folded differently when isolated from mammalian cells [41] compared to bacteria [42], demonstrating that the potential for expression systems to affect protein structure and function is not limited to P35 relatives.

Purification from yeast rather than bacteria resulted in a 280-fold increase in inhibitory activity for MaviP35, but only a six-fold difference for AcP35. Interestingly, SpliP49 proteins purified from insect and bacterial cells were previously reported to be similarly active [27]. Thus, the influence of expression systems on caspase inhibitory activity varies between members of the P35 super-family.

In conclusion, this study revealed that recombinant P35 proteins, especially MaviP35, exhibit stronger activity following purification from yeast than bacteria, suggesting that the true inhibitory potential of some P35 family members may be underestimated from data obtained using bacterially-purified proteins. It is possible that prokaryotic expression systems may also affect the conformation and activity of other classes of proteins normally expressed in eukaryotic cells.

## Materials and Methods

### Plasmid construction

The following plasmids have been previously described: pGALL-(*HIS3*)-AcP35-FLAG [7], pGALL-(*HIS3*)-MaviP35-FLAG [22], caspase 3-pET23a [22]. The 3′ part of AcP35-FLAG was excised from pGALL-(*HIS3*)-AcP35-FLAG with HindIII and XbaI (blunted) and ligated into pET23a-AcP35 [43] cut with HindIII and XhoI (blunted) to generate pET23a-AcP35-FLAG. The MaviP35-FLAG coding region was excised from pGALL-(*HIS3*)-MaviP35-FLAG with NdeI and BamHI and ligated into pET23a to produce pET23a-MaviP35-FLAG.

### Purification and testing of P35 proteins

The *Saccharomyces cerevisiae* yeast strain W303α was transformed [44] and used for protein purification and immunoblotting [22] as previously described. Yeast were grown and transgenes induced at 30°C. The BL21-(DE3)-pLysS strain of *E. coli* (Novagen/Merck, Darmstadt, Germany) was transformed with the pET23a plasmids mentioned above. Caspase 3 was purified as previously described [43]. Expression of FLAG-tagged MaviP35 and AcP35-FLAG were induced with 1 mM IPTG for 6 hours at 30°C. To lyse bacterial pellets, 5 ml BugBuster reagent (Novagen) was used per gram of cells. After 20 min of gentle rocking at RT and centrifugation (36,300 g; 4°C for 15 min), the supernatant was incubated for 30–60 min at 4°C with 300 µl of Anti-FLAG® M2-agarose slurry (Sigma-Aldrich) per liter of culture, which had been previously washed three times with 12× resin volume washing buffer. Phosphate-buffered saline (PBS; Amresco, Solon, Ohio, USA) was used as a wash buffer to prepare samples for analytical ultracentrifugation (AUC); all other gel filtration experiments used 50 mM Tris-HCl pH 7.4, 150 mM NaCl. Following incubation with the lysates, beads were pelleted 5 min at 3,750 g at room temperature, then washed with 32× resin volumes of washing buffer for 10 min at 4°C rotating. Routinely, five elution fractions were collected, each with one resin volume of wash buffer containing 200 ng/µl FLAG peptide (Sigma-Aldrich). For each elution step the beads were incubated for 2–5 min at 4°C with agitation and supernatant was collected after centrifugation. After subsequent SDS-PAGE analysis and Coomassie brilliant blue (Sigma-Aldrich) staining, the fractions containing pure FLAG-tagged proteins were pooled and protein concentration was determined using the BioRad Protein Assay (BioRad) following the manufacturer's protocol. Quantitation of caspase inhibitory activity was performed as previously published [22].

### Crosslinking

Samples were incubated in 5 µM Bismaleimidohexane (BMH; Thermo Scientific, Rockford, IL USA) for 30 min at room temperature prior to boiling in loading dye containing 5% β-mercaptoethanol and analysis by SDS-PAGE and immunoblotting.

### Gel filtration

Gel filtration liquid chromatography was performed using a SuperdexS200 column (GE Healthcare, Little Chalfont, UK). The columns were attached to an ÄKTA purifier (GE-Healthcare) and prepared for loading by washing the column with one matrix volume of water (filtered and degassed via vacuum filtration through a 0.22 µm Durapore membrane (Millipore)) and one volume of filtered, degassed buffer (25 mM ammonium bicarbonate for mass spectrometry; PBS for AUC samples; 20 mM Tris pH 7.4, 50 mM NaCl for other experiments). Subsequently, 250 µl of the protein sample was injected onto the column via a 500 µl injection loop. Size exclusion chromatography was performed for 1.5 column volumes at a steady flow rate of 0.6 ml/min, maximum back pressure of 1.5 MPa and fractions of 500 µl were collected. A buffer sample was collected after completion of protein elution, to use as a control for analytical ultracentrifugation. A protein gel filtration standard (BioRad) was used to determine the elution profile of five proteins (thyroglobulin, bovine γ-globulin, chicken ovalbumin, equine myoglobin, and vitamin B12; MW 1,350–670,000 Da).

### Mass spectrometry

Matrix Assisted Laser Desorption Ionisation-Mass Spectrometry (MALDI-MS) was performed using an Ultraflex III MALDI-tof/tof-MS (Bruker-Daltonics, Germany). The matrix used was 2.5 dihydroxy benzoic acid. A saturated solution of the matrix was obtained by dissolving it in water/acetonitrile of a 7∶3 ratio including 0.1% TFA. The sample was diluted in the same buffer 1∶10 prior to use. Each sample was mixed with the matrix 1∶1 on a steel target plate spot and left to dry. Spectra were acquired until a satisfactory signal to noise level was achieved.

Electrospray Ionisation-Mass Spectro-metry (ESI-MS) was performed using a micrOTOF-Q-MS instrument (Bruker-Daltonics, Germany) linked to an HPLC (Ultimate 3000, Dionex, Holland). Each protein was diluted in 25 mM Tris pH 8.0. DTT (10 mM, pH 8) was added and the peptides were reduced for 1 h at 60°C, then alkylated for 20 minutes at room temperature using a 3 times molar excess of iodoacetamide. Then 0.05 µg of trypsin (Promega) was added and the proteins were digested for 2 h at 40°C. The resulting peptides were injected onto a trapping column (Dionex Acclaim Pepmap100 nanotrap 100 µm×2 cm, C18, 5 µm, 100 Å) using A-buffer (2% acetonitrile in 0.1% formic acid (aq); Sigma-Aldrich). Following a 6 minutes wash the sample was transferred onto a resolving column (Dionex Acclaim Pepmap RSLC, 75 µm×15 cm, C18, 5 µm, 100 Å) and eluted with a gradient of B-buffer (98% acetonitrile in A-buffer) over 70 minutes. The eluent from the column was directly electrosprayed into the mass spectrometer. Mass data was continuously acquired and for each MS spectrum three MS/MS were recorded of the most intense peaks. The data was annotated and deconvoluted using the Data Analysis program (Bruker-Daltonics) and the intensity of the peaks corresponding to all variants of the N-terminal peptide were manually recorded. The sequence of the peptides were confirmed by MS/MS analysis using Biotools and Sequence Editor (Bruker-Daltonics) as well as by manual inspection and peak assignment.

### Circular Dichroism Spectroscopy

Circular dichroism (CD) spectra were recorded using an AVIV Model 410-SF CD spectrometer. Wavelength scans were performed between 190 and 260 nm in 20 mM Tris, 50 mM NaCl pH 7.4 with a sample concentration of 105 µg/ml in 1 mm quartz cuvettes at 20°C. Data collected below 194 nm were removed from analysis due to poor signal-to-noise. To monitor thermostability, thermal denaturation experiments were performed at 0.5°C increments between 20 and 90°C. Data were analyzed using the CDPro software package [35].

### Analytical ultracentrifugation

Sedimentation velocity experiments were conducted in a Beckman model XL-I analytical ultracentrifuge at a temperature of 20°C. All samples were solubilized in PBS. The samples were loaded into a conventional double sector quartz cell and mounted in a Beckman 4-hole An-60 Ti rotor at an initial concentration of 0.28 µM and 0.56 µM for the two bacterial fractions analyzed (denoted 1 and 2 in Figure 6) respectively and 1.95 µM, 2.23 µM and 1.39 µM for the yeast fractions (labeled 1, 2 and 3 in Figure 6), respectively. 380 µl of sample and 400 µl of reference solution were centrifuged at a rotor speed of 40,000 rpm, and the data were collected at a single wavelength (230 nm) in continuous mode, using a step-size of 0.003 cm without averaging. An estimate of the partial specific volume (0.7358 ml/g) was computed using the program SEDNTERP [45]. Sedimentation velocity data at multiple time points were fitted to a continuous sedimentation coefficient [*c(s)*] distribution and a continuous mass [*c(M)*] distribution model [46]–[48] using the program SEDFIT, which is available at www.analyticalultracentrifugation.com.
